# Clinical, Radiological, and Endoscopic Ultrasound Findings in Groove Pancreatitis: A Multicenter Retrospective Study

**DOI:** 10.5152/tjg.2023.22875

**Published:** 2023-07-01

**Authors:** Hussein Hassan Okasha, Mohamed Gouda, Mohammed Tag-Adeen, Mahmoud Farouk, Ahmed Alzamzamy, Sameh Abou Elenin, Katarzyna M. Pawlak, Abeer Awad, Borahma Mohamed

**Affiliations:** 1 Department of Internal Medicine and Hepatogastroenterology, Kasr Al-Aini School of Medicine, Cairo University, Cairo, Egypt; 2 Theodor Bilharz Research Institute, Mouwasat Hospital Dammam, Giza, Egypt; 3 Division of Gastroenterology and Hepatology, Department of Internal Medicine, South Valley University Qena Faculty of Medicine, Egypt; 4 Department of Tropical Medicine and Gastroenterology, Assiut University, Assiut, Egypt; 5 Department of Internal Medicine and Gastroenterology, Maadi Armed Forces Medical Complex, Military Medical Academy, Cairo, Egypt; 6 Division of Internal Medicine, Department of Cardiology, Gastroenterology and Endocrinology, Endoscopy Unit of Hospital of the Ministry of Interior and Administration, Szczecin, Poland; 7 Department of Gastroenterology C, Ibn Sina Hospital, Mohammed V University, Rabat, Morocco; Department of Internal Medicine and Hepatogastroenterology, Kasr Al-aini School of Medicine, Cairo University, Cairo, Egypt

**Keywords:** Groove pancreatitis, pancreatitis, pancreatic head mass, endoscopic ultrasound, EUS-FNA/FNB

## Abstract

**Background/Aims:**

Groove pancreatitis is a rare form of focal pancreatitis that affects the groove area. Since groove pancreatitis may be mistaken for malignancy, it should be considered in patients with pancreatic head mass lesions or duodenal stenosis to avoid unnecessary surgical procedures. The aim of the study was to document the clinical, radiologic, endoscopic characteristics, and treatment outcomes of patients with groove pancreatitis.

**Materials and Methods:**

This retrospective multicenter observational study included all patients diagnosed with one or more imaging criteria suggestive of groove pancreatitis in the participating centers. Patients with proven malignant fine-needle aspiration/biopsy results were excluded. All patients were followed in their own centers and were retrospectively evaluated.

**Results:**

Out of the initially included 30 patients with imaging criteria suggestive of groove pancreatitis, 9 patients (30%) were excluded because of malignant endoscopic ultrasound fine-needle aspiration or biopsy results. The mean age of the included 21 patients was 49 ± 10.6 years, with a male predominance of 71%. There was a history of smoking in 66.7% and alcohol consumption in 76.2% of patients. The main endoscopic finding was gastric outlet obstruction observed in 16 patients (76%). There was duodenal wall thickening in 9 (42.8%), 5 (23.8%), and 16 (76.2%) patients on computed tomography, magnetic resonance imaging, and endoscopic ultrasound, respectively. Moreover, pancreatic head enlargement/mass was observed in 10 (47.6%), 8 (38%), and 12 (57%) patients, and duodenal wall cysts in 5 (23.8%), 1 (4.8%), and 11 (52.4%) patients, respectively. Conservative and endoscopic treatment has achieved favorable outcomes in more than 90% of patients.

**Conclusion:**

Groove pancreatitis should be considered in any case with duodenal stenosis, duodenal wall cysts, or thickening of the groove area. Various imaging modalities, including computerized tomography, endoscopic ultrasound, and magnetic resonance imaging, have a valuable role in characterizing groove pancreatitis. However, endoscopic fine-needle aspiration or biopsy should be considered in all cases to diagnose groove pancreatitis and exclude malignancy, which can have similar findings.

**Main Points**

- Groove pancreatitis should be considered in any case with duodenal stenosis, duodenal wall cysts, or thickening of the groove area.
- The most prevalent clinical and radiologic manifestations in groove pancreatitis are epigastric pain, repeated vomiting, duodenal wall thickening, and the presence of cysts in the duodenal wall or groove area.
- Endoscopic fine-needle aspiration or biopsy should be considered in all cases to diagnose groove pancreatitis and exclude malignancy, which can have similar findings.

## Introduction

**Figure 1.**
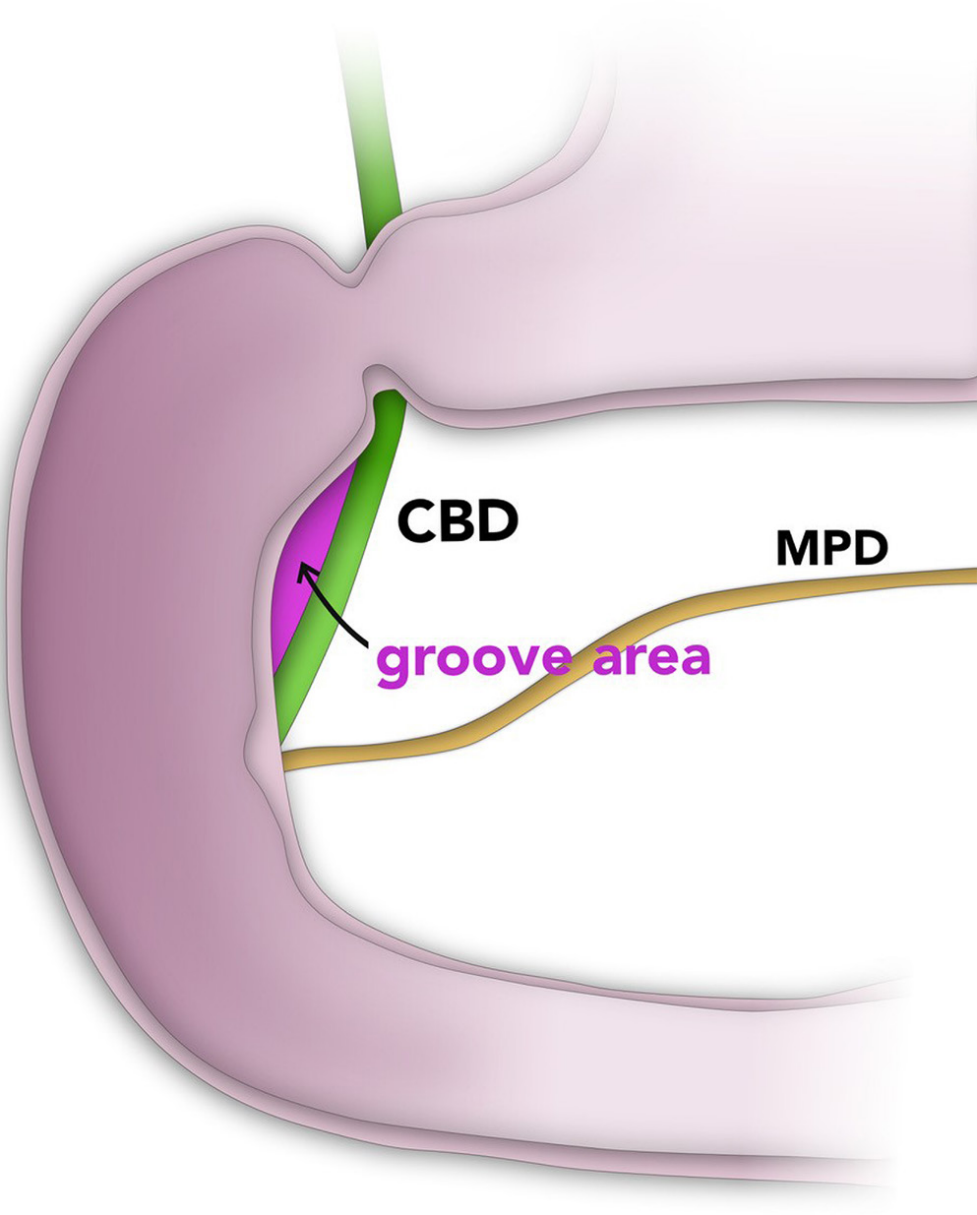
Schematic diagram of the anatomy of the groove area and the “pure” type of groove pancreatitis.

The pancreaticoduodenal groove (PDG) is a small theoretical space bordered medially by the pancreatic head, laterally by the second portion of the duodenum, and superiorly by the duodenal bulb (Figure 1). Several structures are found in this space, including the distal common bile duct (CBD), main pancreatic duct (MPD) of Wirsung, accessory pancreatic duct of Santorini, major papilla, and minor papilla. In addition, many small vessels lie within this space, the most significant of which is the superior pancreaticoduodenal artery and several small lymph nodes.^1^

Groove pancreatitis (GP) is a rare form of pancreatitis that affects PDG.^2^ It is believed to be due to anatomical or functional obstruction of the minor papilla adding on increased viscosity of pancreatic juice by excessive alcohol consumption and/or smoking that could attribute to calcification of the duct. Three types of GP were recognized in the literature; a pure type that involves only the groove area and usually exists early (Figure 1), a segmental type in which the inflammatory process extends to the pancreatic head, and a diffuse type with involvement of the entire pancreas giving the typical picture of chronic pancreatitis. Duodenal mucosa shows thickening, fibrosis, induration, and sometimes cyst formation.^3,4^ Microscopically, GP is characterized by inflammatory cellular infiltrate, Brunner gland and smooth muscle hyperplasia, proteinaceous material surrounded by myofibroblast proliferation, and some degree of cellular atypia that makes the differentiation from malignancy challenging.^5,6^

There is general agreement about a significant relationship between chronic alcohol consumption and the pathogenesis of GP. Pancreatic duct obstruction caused by viscid secretions usually initiates the inflammatory process with subsequent fibrosis and more obstruction with the establishment of a vicious circle of inflammation, fibrosis, and obstruction. This circle is responsible for disease progression, chronicity, and mass formation, resulting in biliary or gastric outlet obstruction (GOO).^7,8^

Imaging plays a crucial role in the diagnosis of GP. Typical computerized tomography (CT) findings of GP include thickening of the medial duodenal wall, ill-defined fat stranding, the frank soft tissue in the groove area, and a duodenal wall cyst. Sometimes, mass-like enlargement of the pancreatic head can be encountered by CT, making the differentiation of GP from pancreatic cancer challenging.^9^ Interestingly, retroperitoneal fat stranding, smudges, and fluid exudation that may be frequently observed in traditional acute pancreatitis are not usually witnessed in GP. However, chronic cases of GP may have similar findings with chronic pancreatitis in the pancreatic parenchyma and ductal systems, such as parenchymal calcifications, ductal dilatation, beading, and irregularity.^10,11^

Magnetic resonance imaging (MRI) and magnetic resonance cholangiopancreatography (MRCP) can reveal the wide distance between the distal part of the ducts and the duodenal lumen caused by duodenal wall thickening and the presence of soft tissue in the groove area. Furthermore, distal CBD and MPD narrowing with upstream dilatation and a dilated banana-shaped gallbladder (GB) can be depicted.^12,13^

The appearance of GP in endoscopic ultrasound (EUS) depends on the stage of the disease and the type of GP. In the early stages, a hypoechoic longitudinal thickening of the PDG is visualized with the thickening of the adjacent duodenal wall and a hypoechoic heterogeneous pancreatic head. In the chronic stages, hyperechoic fibrotic bands are detected in the groove, with hyperechoic thickening of the adjacent duodenum and hyperechoic pancreatic head enlargement. Additionally, it is common to visualize a smooth narrowing of the CBD and the Santorini duct.^14^

Endoscopic ultrasound is currently the tool of choice to evaluate PD groove pathology and groove pancreatitis due to its ability to provide superior visualization of this challenging anatomical region, accessibility, accuracy, and the ability to perform endoscopic fine-needle aspiration or biopsy (EUS-FNA/FNB).^6^

The aim of our study was to document the clinical, radiologic, and endoscopic characteristics and treatment outcomes of patients with GP.

## Materials and Methods

This retrospective multicenter cohort study included all patients diagnosed with GP in the participating centers, including Cairo University Hospital, Egypt; Riyadh University Hospital, Kingdom of Saudi; Assuit University Hospital, Egypt; Maadi Military Hospital, Egypt; Mohammed V University Hospital, Morocco; and Mansoura University Hospital, Egypt.

All patients who had imaging criteria suggestive of GP such as duodenal wall thickening, duodenal wall cyst, soft tissue lesion in the groove area, or pancreatic head enlargement/mass-like lesion were initially included. Patients with proven malignant EUS-FNA/FNB results were excluded from our analysis.

### Study Definitions

Patients were classified into 3 groups according to the type of GP, defined as pure type when the pathology was limited to the groove area, segmental type when it extended medially to the pancreatic head, or diffuse type when it involved the entire pancreas.^3^

### Ethical Approval

The study protocol was conducted following the Helsinki Declaration and approved by the ethical committee board of the Faculty of Medicine, South Valley University, Qena, Egypt, with the reference number: SVUMEDMED0184228432.

### Statistical Analysis

Data on clinical presentation, investigation, operation, and follow-up were analyzed. Variables were reported as mean ± SD for parametric data, median (interquartile) for nonparametric data, and frequencies (percentage) for categorical variables. Statistical analyses were performed using Statistical Package for the Social Sciences (SPSS) version 23.0 (IBM Corp., Armonk, NY, USA).

## Results

A total of 30 patients with one or more imaging criteria suggestive of GP were initially included in this study. However, 9 patients were excluded from our analysis after revising the tissue diagnosis as they had malignant cytology in their FNA/FNB results. Therefore, the study included 21 GP patients with a mean age of 49 ± 10.6 years, 6 females (28.6%), and 14 (66.7%) smokers. Sixteen patients (76.2%) had a history of alcohol intake, 8 of them (38.1%) had a history of continued intake until the diagnosis of GP, with a mean duration of 17.5 ± 4.2 years, while another 8 patients have abstained from alcohol before the first attack of GP. The main presenting symptoms for GP were epigastric pain in 10 patients (47.6%), vomiting in 8 (38.1%), jaundice in 5 (23.8%), and weight loss in 3 (14.3%).

The mean level of CA-19-9 was 87 IU/mL (normal is up to 37 IU/mL). It was not significantly elevated as all cases with proven pancreatic adenocarcinoma were excluded.

All patients were subjected to CT, MRI, and EUS examinations that showed different sensitivity in the detection of GP findings. The significant findings detected by CT, MRI, and EUS were duodenal wall thickening in 9 (42.8%), 5 (23.8%), and 16 (76.2%) patients; pancreatic head enlargement/mass in 10 (47.6%), 8 (38%), and 12 (57%) patients; and duodenal wall cyst in 5 (23.8%), 1 (4.8%), and 11 (52.4%) patients, respectively (Tables 1 and 2). 

**Figure 2.**
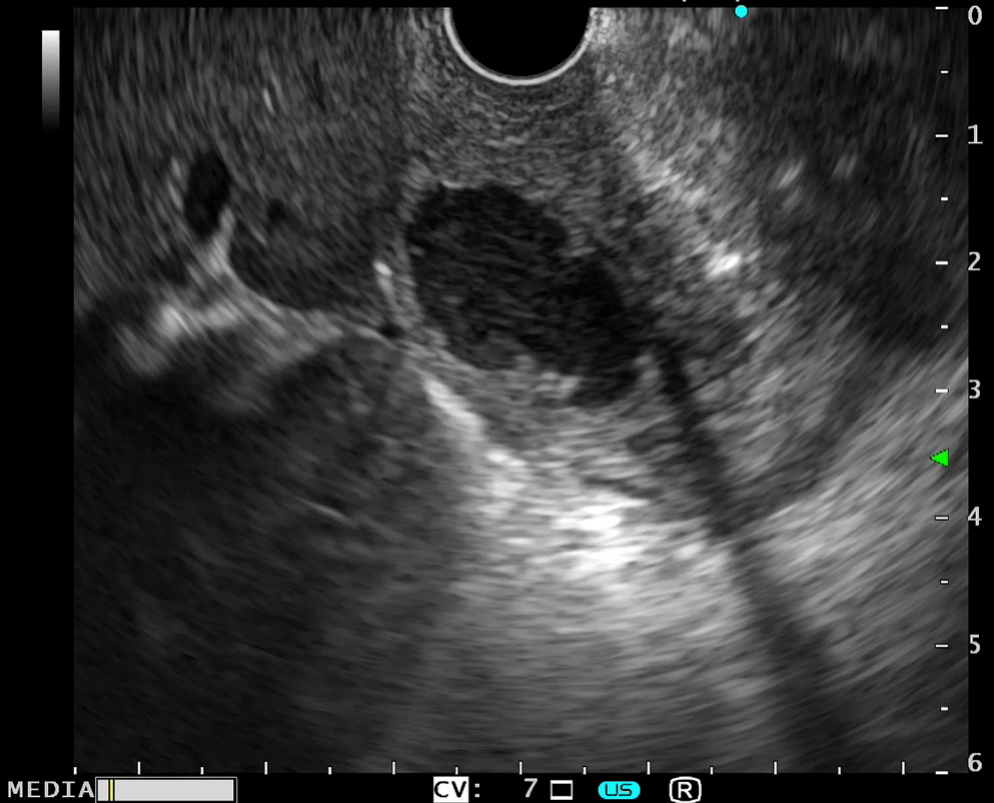
Duodenal wall cyst as seen by endoscopic ultrasound.

In patients with duodenal wall cyst (Figure 2) detected in EUS examination (n = 11), 8 patients had a single cyst and 3 had multiple cysts, with an average cyst size of 7 mm (range: 4-30) (Table 3). All duodenal wall cysts that were detected in the EUS examination were located in the third acoustic layer (the submucosa) of the first or second part of the duodenum, and no cysts were found in the groove area. The cyst content had a slightly turbid echo pattern during the EUS examination (Figure 2). Aspiration of the cyst content was done during EUS mainly for diagnostic purposes, and the aspirate was clear yellowish-brown fluid without any sediments.

Based on the EUS assessment, segmental form of GP was the most prevalent form seen in 14 patients (66.7%), followed by the pure form in 5 patients (23.8%) and the diffuse form in 2 patients (9.5%) (Table 3). There were few peripancreatic lymph node enlargements in all cases, mostly of benign nature as they were small in size (the largest was 4 ×12 mm), echogenic in texture, short/long axis was <1 with preserved hyperechoic hila.

**Figure 3.**
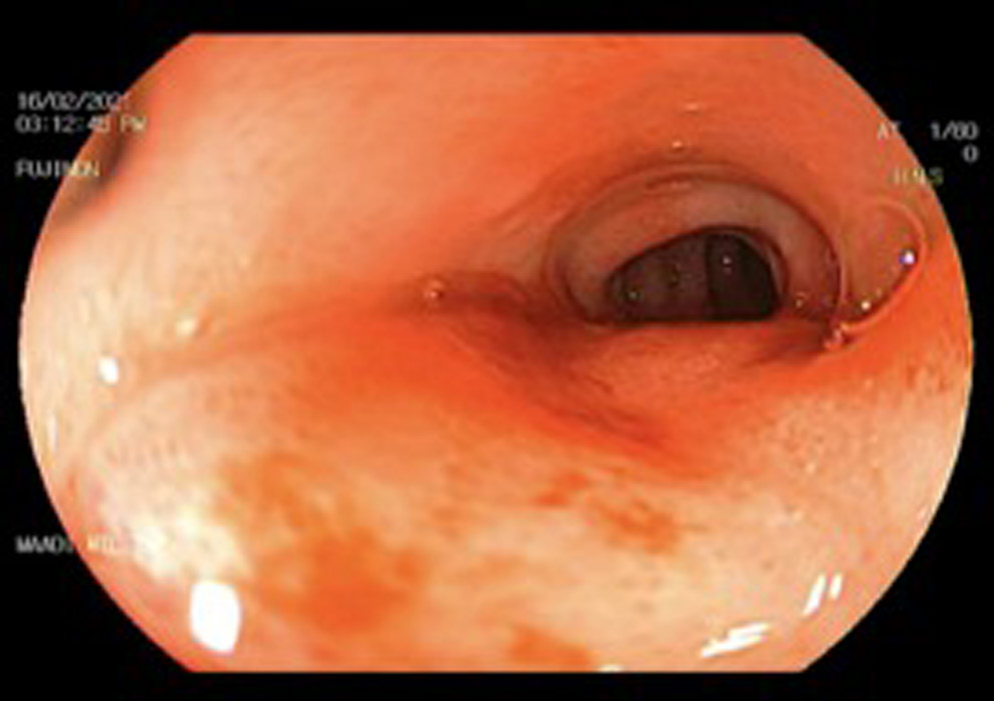
Endoscopic appearance of inflamed edematous duodenal bulb wall.

Endoscopic findings are presented in Table 4 in which 16 patients showed a picture of GOO at different levels: the second part of the duodenum in 10 patients (47.6%), the duodenal bulb in 3 patients (14.3%), and the pylorus in 3 patients (14.3%). Four patients had no significant endoscopic findings, and 1 patient had mucosal congestion and edema in the duodenal bulb (Figure 3). In patients presenting with GOO, endoscopic balloon dilatation was tried successfully; however, 1 case with failed endoscopic balloon dilatation was transferred to surgical gastrojejunostomy.

**Figure 4.**
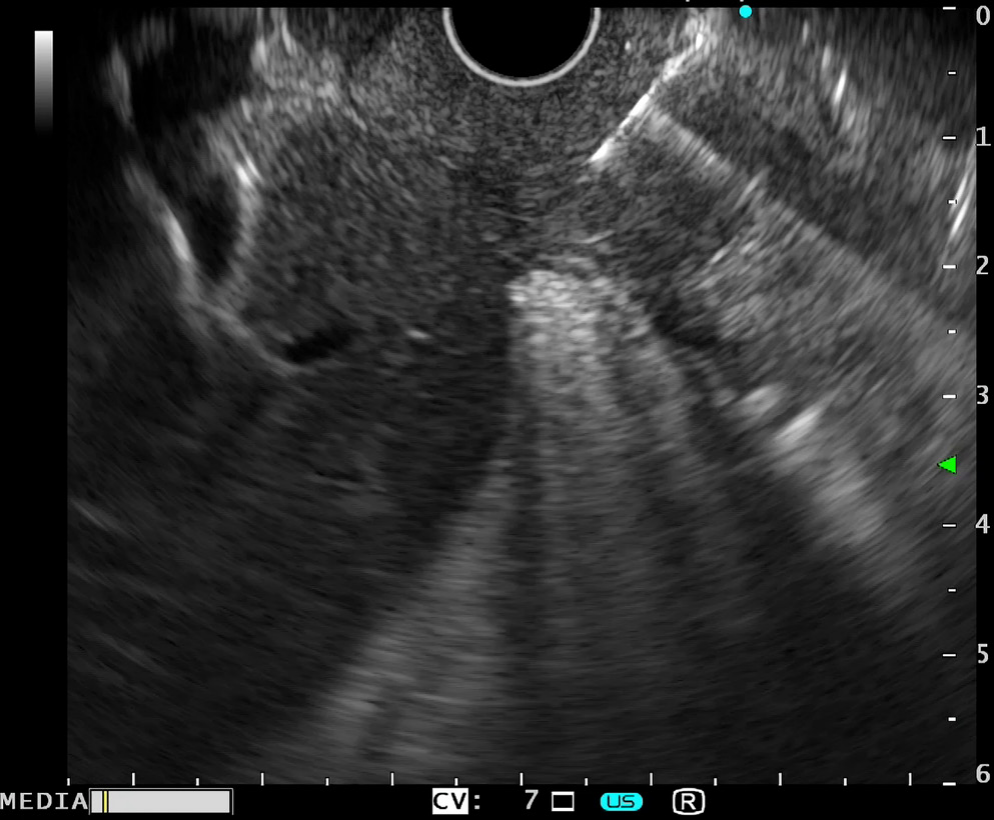
Endoscopic ultrasound fine-needle biopsy of the thickened duodenal wall.

Endoscopic fine-needle aspiration or biopsy (Figure 4) was performed in all patients with its details presented in Table 5. Specimens from the duodenal wall and pancreas were obtained from 9 patients (42.9%) and the duodenal wall from 5 patients (23.8%). The number of EUS sessions required for reaching the definite diagnosis of GP was 1 in 14 patients (66.7%) and more than 1 in 7 patients (33.3%). In patients who required more than 1 EUS session, 2 were scheduled for EUS within 3 months, 4 within 6 months, and 1- within 12-month interval according to the centers’ policy.

Most of the included patients (14 patients, 66.6%) underwent conservative treatment without requiring endoscopic or surgical interventions, and they demonstrated successful outcomes with improved symptoms during a follow-up duration of 6 months.

Six patients (28.5%) underwent endoscopic management, including cyst aspiration (3 patients), plastic CBD stent (1 patient), and MPD stent (1 patient). A plastic biliary stent was inserted in 1 patient who suffered from biliary obstruction with full improvement after its insertion, and then the patient subjected to follow-up and stent exchange after 6-month duration. A pancreatic plastic stent was required in 1 patient with significant MPD dilatation caused by proximal obstruction, the stent was fixed via the major papilla without inner flap to allow its self-falling down after resolution of the inflammatory process. Two patients underwent initial CBD insertion of fully covered self-expandable metal biliary stent (FC-SEMS) followed by a Whipple procedure for 1 of them and gastrojejunostomy for the other patient. Whipple procedure was performed for 1 patient who suffered gastric outlet obstruction caused by GP with failed endoscopic treatment to restore the continuity of the gastrointestinal tract (Table 6).

We reported 2 cases of patient death (9.5%) in our series. Both patients had a severe disease that required surgical intervention. Deaths were related to the disease recurrence and progression and not related to surgery.

## Discussion

Groove pancreatitis is a unique type of pancreatitis occasionally detected in current clinical practice during the radiologic or endosonographic evaluation of pancreatic masses, upper abdominal pain, or pancreatitis. Despite GP sharing nearly the same etiologic factors as conventional pancreatitis, it has a different clinical course with more tendency to recurrence and more significant similarity to the neoplastic process that necessitates surgical excision. Clinically, GP has several symptoms, including epigastric pain, vomiting, jaundice, or weight loss. Most available data about GP are derived from case reports or small case series focusing on the radiologic aspects, mainly CT and MRI, rather than the clinical, endoscopic, EUS, or therapeutic aspects of the disease.

The incidence of GP is not well determined, and most available figures are driven by postoperative histopathologic examination of the surgically excised specimens. A previous study included 160 patients who underwent pancreaticoduodenectomy in a tertiary center in the United Kingdom between 2006 and 2010 for different indications, such as pancreatic cancer, chronic pancreatitis, and carcinoma of the ampulla of Vater. The incidence of GP in the excised surgical specimens was 3% (5/160).^9^ An incidence of 2.6% (8/300) was reported previously by Yamaguchi and Tanaka,^15^ while the incidence in patients who underwent pancreaticoduodenectomy for chronic pancreatitis was relatively higher at 24.3% (30/123 patients).^16^

Clinically, GP is widespread in middle-aged men, with typical symptoms of persistent or recurrent abdominal pain, vomiting, and weight loss. Moreover, a close association between alcohol and tobacco was reported.^17,18^ A retrospective study included 16 GP patients, 12 (75%) males, with a mean age of 58 years (range: 35-73 years). The clinical manifestations in the latter study were epigastric pain in 10 patients, obstructive jaundice in 8, vomiting in 8, weight loss in 6, and diarrhea in 4. Some patients had more than 1 symptom. Mild elevation of amylase and lipase was reported in 8 and 6 patients, respectively, while elevated indirect bilirubin was reported in 8 patients. None of the patients showed a significant rise in the CEA, CA19.9, or other tumor markers.^19^ In agreement with the abovementioned data, our study included 21 patients with a mean age of 49 ± 10.6 years: males represented 72% and smokers represented 66.7%. Regarding alcohol intake, 16 (76%) of the included patients had a history of alcohol intake and 8 patients had a history of ongoing intake until the onset of GP, while the rest abstained from alcohol. Most of our patients showed epigastric pain and vomiting, followed by jaundice and weight loss, which appeared with chronic and recurrent episodes of GP.^20^

Several radiologic findings could be observed in the GP, including fat stranding, hypoenhancing soft tissue in the PDG area, cystic changes in the groove area or the duodenal wall, diffuse pancreatic head enlargement, or mass-like formation, and MPD dilation.^21,22^ The lack of typical radiologic findings for GP makes it challenging to differentiate from pancreatic duct adenocarcinoma (PDAC), particularly in the setting of pancreatic head mass and MPD dilatation. However, some proposed findings were more suggestive of GP, such as cystic changes in the groove area and duodenal wall, thickening of the duodenal wall, and delayed enhancement of the fibrotic tissue involving the groove area. These findings could help avoid surgery for such a benign condition.^22,23^ Fortunately, all patients included in this series were subjected to extensive diagnostic work-up, including CT, MRI/MRCP, and EUS. The most frequently detected findings were duodenal wall thickening, pancreatic head enlargement/mass, duodenal wall cyst, and presence of soft tissue in the PDG area, with the EUS appearing as the most sensitive modality for detection of these findings in comparison to CT and MRI/MRCP.

A significant additional advantage of EUS in assessing suspect cases of GP is the ability to take EUS-FNA/FNB from suspicious lesions. 

Endoscopic fine-needle aspiration or biopsy was obtained from all participants. One session was enough to obtain sufficient specimens in 14 patients (66.7%), while 7 patients (33.3%) required more than 1 EUS-FNA/FNB session. Cytology results showed benign inflammatory aspirate, inflammatory pancreatitis, hemorrhagic and inflammatory aspirate, or desmoplastic atypical cells. As no typical cytology characteristics were reported for GP, the exclusion of malignancy in the obtained cytology and imaging findings and the clinical course were crucial for diagnosis.^6^ Interestingly, the presence of multinucleated giant cells in a cytology specimen obtained from a patient with clinically and radiologically proven groove pancreatitis was previously described.^24^ This variability of the cytology results could be attributed to sampling from different regions or in various stages of the disease and contamination from the duodenal mucosa.

Treatment of GP might include conservative, endoscopic, or surgical options according to disease severity and the presence of gastrointestinal or biliary obstruction. A previous retrospective study compared the outcomes of surgical and nonsurgical management of GP and found that both approaches had similar results in terms of quality of life and pain control. However, a higher incidence of postoperative diabetes was observed with surgical procedures; as a result, appropriate counseling and patient selection are recommended before surgical therapy.^25^ In the current study, the conservative treatment achieved favorable outcomes in 14 patients (66.6%) during a follow-up duration of 6 months. In comparison, 6 patients (28.5%) required endoscopic management, including cyst aspiration and CBD or MPD stenting, and 2 required surgical intervention, either pancreaticoduodenectomy or gastrojejunostomy. One of the patients initially underwent endoscopic stenting (fully covered metal biliary stent) followed by Whipple surgery.

To our knowledge, this is the first multicenter study that included a reasonable number of patients with GP. However, there were certain limitations, such as retrospective design, missing important biochemical data for some patients like pancreatic amylase and lipase levels, and lack of a long-term follow-up.

## Conclusion

The present study showed that alcohol and smoking were the principal risk factors. The typical symptoms were GOO, and the dominant radiologic aspects were duodenal wall thickening and the presence of cysts in the groove. Developments in imaging, especially EUS and MRI, might contribute to the understanding and study of the disease and eliminate the malignancy that represents the most differential diagnosis. However, these imaging modalities should not exclude tissue sampling, which should be carried out in all suspected cases of GP to determine malignancy, the great mimicker of GP. The study showed the most prevalent clinical and radiologic manifestations in GP were epigastric pain, repeated vomiting, duodenal wall thickening, and the presence of cysts in the duodenal wall or groove area. Despite the valuable role of imaging modalities, including EUS, CT, and MRI, in revealing GP, tissue diagnosis should be achieved in suspected cases to rule out malignancy.

## Figures and Tables

**Table 1.** Computerized Tomography Findings Among Patients with Groove Pancreatitis

CT Findings (n = 21)	
Pancreatic head mass-like lesion	6 (28.6)
Pancreatic head enlargement	4 (19)
Duodenal wall thickening	9 (42.8)
Antral wall thickening	1 (4.8)
Soft tissue in the groove area	4 (19.1)
Duodenal wall cyst	5 (23.8)
Pancreatitis features	3 (14.3)

Some patients may have more than one of the above. Results are expressed as frequencies (%).

CT, computerized tomography.

**Table 3.** Endoscopic Ultrasound Findings Among the Patients with Groove Pancreatitis

**EUS findings per patient (n = 21)**	
Duodenal wall thickening	16 (76.2)
Antral wall thickening	2 (9.5)
Maximal wall thickness (mm)	14.8 ± 7.5
Pancreatic head enlargement	12 (57.1)
Diffuse pancreatic enlargement	2 (9.5)
Soft tissue in the groove area	9 (42.9)
Duodenal wall cyst	11 (52.4)
Chronic pancreatitis	4 (19)
CBD dilation	2 (9.5)
PD dilation	2 (9.5)
**Duodenal wall cyst characters in EUS:**	
Single	8 (38%)
Multiple	3 (14.3%)
No cyst	10 (47.6%)
Mean cyst size in mm	7 mm (4 – 30)
**Types of the GP in EUS:**	
Segmental	14 (66.7%)
Pure	5 (23.8%)
Diffuse	2 (9.5%)

EUS**, **endoscopic ultrasound; CBD, common bile duct; GP, groove pancreatitis; PD, pancreatic duct.

**Table 4.** Upper Endoscopic Findings Among Patients with Groove Pancreatitis

Main Endoscopic Finding per Patients (n = 21)	
GOO at the level of D2	10 (47.6%)
GOO at the level of duodenal bulb	3 (14.3%)
GOO at the level of pyloric ring	3 (14.3%)
Congested and erythematous bulbar mucosa	1 (4.8%)
Non-significant endoscopic findings	4 (19%)

GOO, gastric outlet obstruction.

**Table 5.** Details of EUS-FNA/FNB Procedures in the Included Groove Pancreatitis Patients

1. Site of sampling	
Duodenal wall and pancreas	9 (42.9)
Duodenal wall	5 (23.8)
Duodenal wall and groove area	4 (19)
Cyst wall	3 (14.3)
2. Type of needle	
FNA 22 G	8 (38.1)
FNA 19 G	3 (14.3)
FNB 22 G	9 (42.9)
FNB 20 G	1 (4.8)
3. Number of sessions of EUS-FNA/FNB required	
One session	14 (66.7)
Two sessions	2 (9.5)
Three sessions	4 (19)
Four sessions	1 (4.8)
4. Results of sampling*	
Benign inflammatory aspirate	15 (71.5)
Inflammatory pancreatitis	8 (19)
Hemorrhagic and inflammatory	2 (9.5)
Desmoplastic atypical cells	1 (4.8)
Follow-up duration (months)	6 (3-12)

*Some patients have more than 1 cytology results.

EUS-FNA/FNB, endoscopic ultrasound fine-needle aspiration/fine-needle biopsy.

**Table 6.** Clinical Outcomes after Conservative, Endoscopic, and Surgical Treatment

Type of treatment (n = 21)*		Success	Failure
1. Conservative (n = 14)		14 (66.7)	
2. Endoscopic (n = 6)		5 (23.1)	1 (4.8)
Aspiration	3		
CBD plastic stent	1 (4.8)				
CBD FC-SEMS	2				
PD plastic stent	1 (4.8)				
3. Surgery (n = 2)		2	
Whipple	1 (4.8)		
Gastrojejunostomy	1 (4.8)		

*Some patients may be subjected to more than 1 therapeutic modality.

CBD, CBD, common bile duct; FC-SEMS, fully covered self-expandable metal biliary stent; PD, pancreatic duct.

**Table 2.** Magnetic Resonance Imaging Findings Among the Patients with Groove Pancreatitis

MRI Findings (n = 21)	
Pancreatic head mass-like lesion	5 (23.8)
Pancreatic head enlargement	3 (14.3)
Duodenal wall thickening	5 (23.8)
Antral wall thickening	1 (4.8)
Duodenal wall cyst	1 (4.8)
CBD dilation	1 (4.8)
PD dilation	1 (4.8)
Narrowing duodenal lumen	1 (4.8)

Some patients may have more than one of the above. Results are expressed as frequencies (%).

CBD, common bile duct; MRI, magnetic resonance imaging; PD, pancraetic duct.
